# Intratumorally injected pro-inflammatory allogeneic dendritic cells as immune enhancers: a first-in-human study in unfavourable risk patients with metastatic renal cell carcinoma

**DOI:** 10.1186/s40425-017-0255-0

**Published:** 2017-06-20

**Authors:** Anna Laurell, Maria Lönnemark, Einar Brekkan, Anders Magnusson, Anna Tolf, Anna Carin Wallgren, Bengt Andersson, Lars Adamson, Rolf Kiessling, Alex Karlsson-Parra

**Affiliations:** 1 Department of Oncology, Uppsala University Hospital, Uppsala, Sweden; 2 Department of Surgical Sciences, Radiology, Uppsala University Hospital, Uppsala, Sweden; 3 Department of Surgical Sciences, Urology, Uppsala University Hospital, Uppsala, Sweden; 4 Department of Immunology, Genetics and Pathology, Uppsala University Hospital, Uppsala, Sweden; 5 Department of Pathology, Karolinska Institutet, Stockholm, Sweden; 6 Department of Microbiology and Immunology, University of Gothenburg, Gothenburg, Sweden; 7 Department of Oncology-Pathology, Karolinska Institutet, Stockholm, Sweden; 8 Immunicum AB, Gothenburg, Sweden

**Keywords:** Metastatic renal cell carcinoma, Phase I/II study, Intratumoral administration, Vaccine, Allogeneic dendritic cells, Anti-tumor response, INTUVAX, Sunitinib

## Abstract

**Background:**

Accumulating pre-clinical data indicate that the efficient induction of antigen-specific cytotoxic CD8+ T cells characterizing viral infections is caused by cross-priming where initially infected DCs produce an unique set of inflammatory factors that recruit and activate non-infected bystander DCs. Our DC-based immunotherapy concept is guided by such bystander view and accordingly, we have developed a cellular adjuvant consisting of pre-activated allogeneic DCs producing high levels of DC-recruiting and DC-activating factors. This concept doesn’t require MHC-compatibility between injected cells and the patient and therefore introduces the possibility of using pre-produced and freeze-stored DCs from healthy blood donors as an off- the-shelf immune enhancer. The use of MHC-incompatible allogeneic DCs will further induce a local rejection process at the injection site that is expected to further enhance recruitment and maturation of endogenous bystander DCs.

**Methods:**

Twelve intermediate and poor risk patients with newly diagnosed metastatic renal cell carcinoma (mRCC) where included in a phase I/II study. Pro-inflammatory allogeneic DCs were produced from a leukapheresis product collected from one healthy blood donor and subsequently deep-frozen. A dose of 5–20 × 10^6^ DCs (INTUVAX) was injected into the renal tumor twice with 2 weeks interval before planned nephrectomy and subsequent standard of care.

**Results:**

No INTUVAX-related severe adverse events were observed. A massive infiltration of CD8+ T cells was found in 5 out of 12 removed kidney tumors. No objective tumor response was observed and 6 out of 11 evaluable patients have subsequently received additional treatment with standard tyrosine kinase inhibitors (TKI). Three of these 6 patients experienced an objective tumor response including one sunitinib-treated patient who responded with a complete and durable regression of 4 brain metastases. Median overall survival (mOS) is still not reached (currently 42.5 months) but has already passed historical mOS in patients with unfavourable risk mRCC on standard TKI therapy.

**Conclusions:**

Our findings indicate that intratumoral administration of proinflammatory allogeneic DCs induces an anti-tumor immune response that may prolong survival in unfavourable risk mRCC-patients given subsequent standard of care. A randomized, multi-center, phase II mRCC trial (MERECA) with INTUVAX in conjuction with sunitinib has been initiated.

**Trial registration:**

Clinicaltrials.gov identifier: NCT01525017.

## Background

Despite the clinical improvement with VEGF and mTOR-targeted therapies in improving clinical outcomes in mRCC, resistance to these therapies develops in the vast majority of cases and despite of considerable efforts in sequential or combined modalities, durable remissions are rare. These facts have resulted in continued interest in novel immunomodulating agents for the treatment of mRCC. Notably, in a recent phase III trial with the immune checkpoint inhibitor nivolumab (a human monoclonal antibody targeting programmed cell death protein (PD-1)) given to patients with previously treated advanced RCC, overall survival was significantly increased when compared to standard second-line treatment with everolimus [1]. Based on these results, nivolumab received approval from the FDA in November 2015 for the treatment of advanced RCC in patients who have received prior antiangiogenic therapy.

Mutation-derived and tumor-specific neoantigens are now increasingly recognized as immunodeterminants, as there is strong evidence that neoantigen-specific T cells are reactivated, following treatment with checkpoint blockade rendering them capable of mediating tumor rejection [2]. These results reveal that tumor-specific neoantigens are not only important targets of checkpoint blockade therapy, but can also be used to develop personalized cancer-specific vaccines [3]. To exemplify, this strategy was used successfully by Carreno et al. in stage 3 melanoma patients by promoting a diverse neoantigen-specific T cell response [4]. Vaccination using autologous dendritic cells (DCs) loaded with patient-specific neoantigens was found to be safe, and none of the patients had evidence of autoimmunity. However, with this type of personalized DC-based vaccines, tumor tissue from each patient is required to identify and prioritize candidate neoantigens, which implicate coordination of tissue acquisition, collection of an autologous leukapheresis product, construction of a vaccine, and administration of the produced vaccine [5].

One obvious way to circumvent practical problems associated with ex vivo production of a complete neoantigen-based vaccine would be to use the patient’s own tumor in situ, as a direct antigen source by intratumoral administration of a potent immune enhancer. The immune-enhancing role would be to recruit and activate the patient’s endogenous DCs and DC precursors to the tumor site, where they will encounter and engulf dying tumor cell and/or tumor cell debris, including the full array of “personal” mutation-derived neoantigens.

As to potential potent immune enhancers, recent studies have shown that injected autologous DCs, which have been pre-activated during a limited time period ex vivo, have the potential to indirectly prime naïve CD8+ T cells in vivo, by acting as a pure immunogenic adjuvant and not as antigen presenters [6, 7]. This indirect immune enhancing function of DCs is strictly dependent on their active secretion of certain DC-and-NK-cell-recruiting chemokines at the time of administration [6]. In line with these data, pre-clinical findings indicate that the efficient induction of antigen-specific cytotoxic CD8^+^ T lymphocytes (CTLs), characterizing viral infections, is caused by cross-priming, where initially infected DCs produce a unique set of inflammatory factors that recruit and activate non-infected “bystander” DCs [8–11].

In vitro studies, using human monocyte-derived DCs, have further shown than these cells can be programmed to produce vigorous amounts of T helper 1-associated chemokines and cytokines in a sustained fashion when stimulated with a combination of stimulatory factors, including Toll-like receptor (TLR) ligands and IFN-gamma, thus mimicking virally infected DCs [12, 13]. Moreover, in the study by Pang et al., using transgenic mice, it was shown that no MHC compatibility was needed between infected DCs and “bystander” DCs [10]. By using allogeneic DCs as immune enhancers in the vaccine setting, such cells will further be regarded as foreign allogeneic invaders that most likely will potentiate a T helper 1-deviated inflammatory reaction, further promoting recruitment and activation of endogenous “bystander” DCs at the vaccination site [14].

If the pro-inflammatory DCs are injected intratumorally, tumor-derived proteins (including tumor-specific neoantigens) will act as an antigen source for recruited endogenous “bystander” DCs. The final outcome of the proposed scenario is that recruited endogenous DCs will capture dying tumor cells and subsequently become activated into T helper 1 deviating DCs. These DCs will finally migrate to the tumor-draining lymph node, where they will prime tumor-specific T cells, including cytotoxic CD8+ T cells [6]. In line with this proposed scenario, supernatants from activated human monocyte-derived DCs (activated with a cocktail consisting of R848, poly I:C and IFN-gamma) have been shown to induce a phenotypic maturation of “bystander” immature DCs in vitro (15). Also, intratumorally or subcutaneously injected allogeneic bone-marrow derived mouse DCs, activated with an identical cocktail (except that human IFN-gamma has been replaced by mouse IFN-gamma), have been shown to both induce recruitment of immune cells into the vaccination site and migration of mature endogenous DC to the draining lymph node. Moreover, by loading subcutaneously injected allogeneic mouse DCs with a tumor antigen, a systemic activation of tumor-specific T cells was further demonstrated [15].

Here, we present safety as well as immunological and efficacy outcomes from the first-in-human study in poor and intermediate risk patient with newly diagnosed mRCC, receiving 5–20 × 10^6^ DCs (INTUVAX) by a CT-guided injection into the renal tumor twice with 2 weeks interval before planned nephrectomy and subsequent standard of care.

## Methods

### INTUVAX production

INTUVAX was manufactured at the Good Manufacturing Practice (GMP) compliant facility of the Cancer Centre Karolinska (CCK), Stockholm, Sweden. Monocytes were isolated from a leukocyte concentrate from a healthy blood donor at the Blood Centre, Karolinska University Hospital. The monocytes were cultivated in CellGro® DC Medium (CellGenix™), a serum free cell culture medium, supplemented with 100 ng/mL granulocyte macrophage colony-stimulating factor (GM-CSF) and 20 ng/mL interleukin 4 (IL-4) (both cytokines from CellGenix™). Thereafter, the cells were cultured in a closed system and incubated at 37 °C in 5% CO_2_ atmosphere. After differentiation, the CellGro® DC Medium was supplemented with 100 ng/mL GM-CSF and 20 ng/mL IL-4 and added to the cell suspension. To induce DC activation/maturation, toll-like receptor (TLR) 7/8 agonist R848 (2.5 μg/mL; InvivoGen), TLR3 agonist Poly I:C (20 μg/mL; Sigma-Aldrich), and human recombinant interferon gamma (IFN-γ; Imukin) (1000 U/mL; Boeringer-Ingelheim) were added to the culture medium. After maturation of DCs, mature pro-inflammatory DCs were harvested and resuspended in heat-inactivated AB plasma obtained from the Blood Centre, Karolinska University Hospital, and supplemented with 10% DMSO. One mL of the cell suspension was filled in each cryovials (CryoTubes™, NUNC) and subsequently deep-frozen to −150 °C, using a computerized gradient freezer (Planer) and thereafter transferred to a − 150 °C freezer. The final product, INTUVAX, was thawed and assessed for release tests (including testing for sterility, mycoplasma and endotoxin) for clinical use. All doses of INTUVAX that were used in this study originate from the same batch.

### Patients

Men and women at least 18 years of age with newly diagnosed synchronous mRCC were enrolled. Inclusion required a size of the primary kidney tumor of at least 4 cm in longest diameter, as determined by CT, and at least one measurable distant metastatic lesion. Patients should have an Eastern Cooperative Oncology Group (ECOG) performance status <3 and were required to be candidates for nephrectomy and to have adequate hematological parameters. Therefore, patients with a life expectancy of less than 3 months, brain metastases, active or latent virus disease (HIV, HBV and HCV), other malignancy or ongoing active autoimmune disease, which required treatment with systemic immunosuppressive agents were excluded.

### Study design and treatment

The study was a prospective single armed, open label phase I/II study. Patients on the waiting list for nephrectomy were enrolled in 3 cohorts of 3–5 patients per cohort. All patients in the same cohort were treated at the same dose level, i.e. 5 × 10^6^, 10 × 10^6^ or 20 × 10^6^ viable and MHC class II expressing DCs. Two CT-guided injections with 14 ± 3 days of interval were administered into the viable part of the primary renal tumor identified with contrast enhanced CT scan. All INTUVAX doses used in the study were from the same batch.

The planned nephrectomy was performed within 28–42 days after the first vaccination. The patients were hospitalized for 24 h after each vaccination. The patients then returned to the clinic for safety visits one week after each vaccination, in connection with nephrectomy and 3 months after nephrectomy. Monitoring of vital signs, physical examination, safety lab and collection of adverse events (graded according to the National Cancer Institute Common Toxicity Criteria (CTC) version 4.0), were made at visits during the treatment period and at the follow-up visit, 3 months after nephrectomy.

Immunologic parameters including inflammatory immune serology, immune cell parameters, INTUVAX cell tracking and analyses using enzyme-linked immunosorbent spot (ELISpot) were monitored in connection with the vaccinations (samples taken at the day for vaccination and the day after). To evaluate potential auto- and allo-immune events, auto- and allo-immunization parameters were evaluated at screening and at follow-up visit 3 months after nephrectomy. Information on disease progression and survival data were obtained for all patients. Tumor follow-up, according to Response Evaluation Criteria In Solid Tumors (RECIST) 1.1 criteria, was conducted at baseline and at follow-up visit 3 months after nephrectomy. An extended follow-up to evaluate clinical outcome was further performed.

### Study oversight

This study was approved by the local ethics committee in Uppsala, Sweden, and was conducted in accordance with Good Clinical Practice guidelines, as defined by the International Conference on Harmonisation. All patients provided written informed consent that was based on the principles of the Declaration of Helsinki. A safety monitoring committee reviewed safety during this study. The study was designed by the authors in collaboration with the sponsor (Immunicum AB, Gothenburg, Sweden).

### End points

The primary endpoints were registration of adverse events and changes in vital signs and laboratory parameters from baseline. Secondary end points included evaluation of tumor-specific immune response using immunohistology parameters of the renal tumor post nephrectomy, tumor-specific ELISpot assay, inflammatory immune parameters, systemic cytokine and chemokine production, immune cell distribution and activation, vaccine cell tracking and evaluation of potential auto- and allo-immunization. Finally, tumor response by CT scan was evaluated, according to RECIST 1.1.

### Blood sample collection and storage

Heparinized blood was drawn before treatment, during the vaccinations, and at the end of the study. Peripheral blood mononuclear cells (PBMCs) were isolated by centrifugation on Metrizoate-ficoll (Lymphoprep, Nycomed/Axis-Shield PoC/Alere Technologies AS, Oslo, Norway) using a standard method, and thereafter aliquoted and cryopreserved for later use. Serum samples were also collected before treatment and at the end of the study and stored at −80 °C until analysis.

### HLA-typing

At screening, evaluation of the patient’s HLA-type (HLA-A, HLA-B and HLA-DR) was made. The samples were analyzed at the local clinical immunology laboratory at site (Dept of Clinical Immunology, Uppsala University Hospital, Uppsala, Sweden). HLA-typing of vaccine cell donor was also performed.

### Tracking of injected INTUVAX cells

Flow cytometry analysis for donor cell tracking was performed on peripheral blood samples (taken just before vaccination and 1 h and 20–24 h after each vaccination) after lyzing of the erythrocytes with ammonium chloride and washing using a standard procedure. The samples were stained with combinations of murine monoclonal antibodies directly conjugated with fluorochromes (fluorescein isothiocyanate; FITC, phycoerythrin; PE, peridinin-chlorophyll protein; Per-CP or allophococyanin; APC). Staining was performed with antibodies specific for HLA class I antigens (HLA-A2 or HLA-A24) selectively expressed on INTUVAX cells, HLA-DR and CD86. The cells were incubated at 4 °C for 15 min with antibodies in the concentrations recommended by the manufacturer. The cells were analyzed in a FACSCanto™ II flow cytometer using the FACSDiva software and dot plots and quadrant statistics from a four-colour analysis were generated. All antibodies, the flow cytometer and the software were from BD Biosciences (Mountain View, CA, USA). Results for each subpopulation were expressed as the percentage of leucocytes. The samples were analyzed at the Dept of Clinical Immunology, Sahlgrenska University Hospital, Gothenburg, Sweden.

### Serologic inflammatory parameters

Cytokines and chemokines were quantified from serum samples taken just before and 1 day after each administration of INTUVAX cells and 14 days after the second administration using a Bio-Plex human cytokine assay, a Bio-Plex 100 assay reader and the Bio-Plex Manager™ software (Bio-Rad Laboratories AB, Sundbyberg, Sweden). The analysis was performed according to the manufacturer’s instructions. The following inflammatory parameters were analyzed: IL-1 beta, IL-2, IL-4, IL-5, IL-6, IL-7, IL-8, IL-10, IL-12p70, IL-13, IL-17A, G-CSF. GM-CSF, IFN-gamma, MCP-1, MIP-1 beta and TNF-alpha. The chemokine RANTES was quantified by a commercially available ELISA (R&D Systems, Abingdon, UK), according to the manufacturer’s instructions. The production of TNF-alpha, IL-1 beta, IL-12p70, RANTES and MIP-1 beta was also analysed in supernatants from thawed and washed Intuvax cells cultured for 24 h in AIM-V culture medium at a concentration of 1 × 10^6^ cells/mL. The samples were analyzed at the Dept of Clinical Immunology, Sahlgrenska University Hospital, Gothenburg, Sweden.

### Lymphocyte distribution in peripheral blood

In order to evaluate potential changes in lymphocyte subset occurrence and activation state in peripheral blood taken just before and 1 day after each administration of INTUVAX cells and 14 days after the second administration, the following parameters were analyzed by FACS, as described above. Antibodies to the following antigens were used: CD3, CD4, CD8, CD19, CD16, CD56 and HLA-DR. The absolute number of blood lymphocytes was determined with Trucount reference beads using the method recommended by the manufacturer. The following subpopulations were reported CD3+, CD3 + 4+ and CD3 + 8+ T cells, CD3 + HLA-DR+ activated T cells, CD19+ B cells, CD3–16 + 56+ NK cells and CD3 + 16 + 56+ NKT cells. The results for each subpopulation were expressed as the percentage of lymphocytes and as the number of cells × 10^9^/L. All antibodies and the Trucount beads were from BD Biosciences.

### Immunohistochemistry

A randomly selected tissue specimen from the renal tumor was formalin-fixed, paraffin-embedded and cut into 5 μm sections. The sections were deparaffinised, and rehydrated in a graded series of ethanols. Antigen retrieval was done by heating tissue sections, using a Target Retrieval Solution, pH 9.0 (DAKO). The samples were subsequently incubated for 5 min in peroxidase blocking solution (DAKO) to inhibit endogenous peroxidase. Consecutive sections from each tumor specimen were then incubated with antibodies against CD3 (polyclonal rabbit anti-human CD3; DAKO), CD4 (monolonal mouse anti-human CD4, clone 4B12; DAKO), CD8 (polyclonal rabbit anti-human CD8; DAKO), CD56 (monoclonal mouse anti-human CD56, clone 123C3; DAKO), CD68 (monoclonal mouse anti-human CD68, clone KP1; DAKO), HLA-DR (monoclonal mouse anti-human HLA-DR, alpha chain, Clone TAL.1B5; DAKO) and PD-L1 (monoclonal mouse anti-human PD-L1, Clone 22C3, DAKO-kit SK006, DAKO) for 30 min. A subsequent reaction was carried out using secondary antibodies (DAKO) at 37 °C for 30 min. Then, the sections were washed three times with phosphate-buffered saline and subsequently the color was displayed with DAB (DAKO) for about 5 min. Sections were counterstained with haematoxylin, dehydrated in ethanol, cleared in xylene, and coverslipped.

For enumeration of CD8-infiltrating T cells, five different areas with most abundant positively stained cells in each section were selected and number of positve cells were counted under 400 magnification [16]. The median number in each sample was then calculated. A semi-quantitative evaluation of infiltrating CD3+ T cells, CD4+ T cells, CD56+ NK/NKT cells, CD68+ macrophages and HLA-DR expressing non-tumor cells was performed. Moreover, the expression of HLA-DR by tumor cells was assessed.

### ELISpot

The potential tumor-specific T cell response, induced by INTUVAX treatment was evaluated by running a tumor-specific IFN gamma (IFN-γ) ELISpot. Blood samples were collected at day 1 (before first vaccination) and 2 weeks after the second vaccination (in connection with hospitalization for nephrectomy). PBMC were isolated and frozen for subsequent batched analysis.

Fresh autologous tumor material from the resected tumor was deep-frozen (−70 °C) and subsequently thawed, homogenized with a GenltleMACS tissue homogenizer (Miltenyi Biotec GmbH, Bergisch Gladbach, Germany), followed by six cycles of freezing at −130 °C and thawing. After centrifugation and sterile filtration of the supernatant, the method of Bradford was used to determine protein concentration. The lysate was used as an antigenic source in the tumor-specific ELISpot assay.

Freshly drawn PBMCs from each patient were used for differentiation of immature monocyte-derived DCs, as described above for the production of INTUVAX. Immature DCs were subsequently pulsed with the tumor material (100 μg/mL) described above and stimulated for 24 h with a cocktail identical with the one used for INTUVAX production.

A direct ex vivo interferon gamma ELISpot analysis was then performed, using the commercial Human IFN-γ ELISpot^PLUS^ kit (MABTECH AB, Nacka Strand, Sweden), according to instructions from the manufacturer.

Briefly, one vial of frozen PBMCs from each time point was thawed, counted in a cell counter (Sysmex K-4500; TOA Medical Electronics Co, Japan) and plated into the wells of an anti-IFN-γ antibody (clone 1-D1K, Mabtech, Inc) pre-coated ELISpot plate at 100,000 and 200,000 cells per well. Cells were then stimulated with 20,000 tumor-loaded autologous mature DCs per well for 24 h at 37 °C. As a positive control, an antibody to CD3 was used. After 24 h, plates were washed and incubated with a biotinylated anti-IFN-γ secondary antibody (clone 7-B6–1, Mabtech, Inc) for 2 h at room temperature. The plates were washed and incubated with streptavidin-conjugated alkaline phosphatase for 1 h and then washed again, and incubated with substrate for alkaline phosphatase. Excess substrate was removed by rinsing with tap water. The number of spots were counted using a dissection microscope. All samples were analyzed in triplicates, and the mean response (spots per 100,000 cells per well) was calculated. The analyses were performed at the Department of Clinical Immunology, Sahlgrenska University Hospital, Gothenburg, Sweden.

### Measurement of potential auto- and alloimmunizaton

To evaluate potential autoimmune events, screening for autoantibodies against clinically relevant autoantigens, including nuclear antigens (ANA, SSA, SSB, Sm, RNP, Scl-70, Centromeres and Jo-1) and kidney parenchyma-associated autoantigens (liver-kidney microsomal antigens and mitochondrial antigens) was made. Samples were taken at screening and at day 120. The samples were analyzed at the Dept. of Clinical Immunology, Uppsala University Hospital, Uppsala, Sweden.

To evaluate potential vaccine-induced alloimmunization at the humoral level, screening for alloantibodies against HLA-A, B, C (MHC-class I) and HLA-DR, DQ, DP (MHC-class II) antigens was made. Samples were taken at screening and at day 120. The samples were analyzed at the Dept of Clinical Immunology at Uppsala University Hospital.

### Tumor response

Tumor response was evaluated by CT scan, comparing the baseline CT scan (obtained within 28 days before day 1) with the CT scan made 3 months’ post nephrectomy, according to RECIST 1.1 criteria [17].

## Results

### Patients

From January 2012 through August 2013, 12 newly diagnosed mRCC patients were enrolled at Uppsala University Hospital, Uppsala, Sweden. At the time of inclusion, all 12 patients were assessed as RCC with at least one metastasis. The RCC diagnosis was based on CT scan and pretreatment biopsy for histopathological verification was not performed. The RCC diagnosis was, however, confirmed by histopathology of the nephrectomy specimen in all patients. Eleven patients had clear cell RCC, while one patient had papillary RCC (patient 5). Demographic and Disease Characteristics are presented in Table 1.

**Table 1:** Demographic and disease characteristics

Characteristics/Variable	No. of patients (%)
Age	
Median	61.5
Range	49–81
Sex	
Female	2 (16.7)
Male	10 (83.3)
ECOG (screening)	
Grade 0	10 (83.3)
Grade 1	2 (16.7)
Time from diagnosis to treatment	1.0 month (Mdn)
Metastatic disease	11 (91.7)
Histologic subtypes	
Clear cell	11 (91.6)
Non-clear cell (papillary)	1 (8.4)
Sarcomatoid features	6 (50)
Fuhrman nuclear grade	
Grade 1	2 (16.7)
Grade 3	7 (58.3)
Grade 4	3 (25.0)
MSKCC [38] and/or IMDC [39] variables	
< 1 year from diagnosis to targeted treatment	11 (100)
Karnofsky performance status <80%	1 (9.1)
Haemoglobin < LLN	8 (72.7)
Serum corrected calcium conc. > ULN	5 (45.5)
Lactate dehydrogenase >1.5 x ULN	1 (9.1)
Neutrophil count > ULN	0 (0)
Paltelet count > ULN	3 (27.2)
MSKCC prognostic group	
Favourable	0 (0)
Intermediate	5 (45.5)
Poor	6 (54.5)
IMDC prognostic group	
Favourable	0 (0)
Intermediate	6 (54.5)
Poor	5 (45.5)

*Abbreviations: LLN* lower limit of normal, *ULN* upper limit of normal, *MSKCC* Memorial Sloan-Kettering Cancer Center, *IMDC* International Metastatic RCC Database, *Mdn* median

One patients (patient 6) with RCC and presumed RCC bone metastasis was later found to have multiple myeloma. This was not suspected until the patient developed progressive detoriation of the renal function, due to light chain damage. Retrospective analysis of the plasma proteins showed that myeloma was present before enrollment in this study. All 12 patients were included in both the efficacy and safety sets. However, patient 6 was not included in evaluation of tumor response or survival as the patient had 2 concomitant cancer diseases and thus not RCC with metastases.

### INTUVAX characteristics and patient exposure

The manufactured INTUVAX batch passed quality (release) tests according to GMP guidelines, including sterility and endotoxin level (< 5 EU/mL). Number, viability, and HLA-DR expression was evaluated directly after thawing and the total number of viable and HLA-DR expressing cells/vial was found to be 12,6 million cells. The production of TNF-alpha, IL-1 beta, IL-12p70, MIP-1 beta and RANTES measured 24 h after thawing was 300, 800, 7.870, 6.460 and 29.000 pg/mL, respectively. When acceptance criteria (data not shown) of thawed INTUVAX cells were met, the rest of the frozen vials in the actual batch were transported from the CCK GMP laboratory to Vecura Clinical Research Center, Karolinska University Hospital, Stockholm, and transferred to a − 150 °C freezer for cell banking until time for vaccination.

On the day of administration/the day before (maximum 24 h before administration), the frozen vial was sent on dry ice to the local hospital pharmacy, where the final preparation of the INTUVAX product was made. Immediately before administration to the patient, the cells were thawed, washed and resuspended into final concentration of 10 or 20 × 10^6^ cells/mL in 0.15 M saline (Sodium Chloride; Braun Medical Inc.) with 2% human serum albumin (Albunorm; Octapharma). The estimated residual amount of R848, poly-I:C and IFN-gamma in each dose of the resuspended drug product was 0.25 ng, 2 ng and 0.1 Units, respectively. The administration of INTUVAX was performed within 1 h from thawing. All patients received 2 intratumoral administrations as per protocol. Patients 1–4 received 5 × 10^6^ cells in 0.5 mL/dose, patients 5–9 10 × 10^6^ cells in 0.5 mL/dose and patient 10–12 20 × 10^6^ cells in 1.0 mL/dose.

### Safety

In terms of AEs (Table 2), the probably or possibly drug-related events reported were fever (5 patients) and chills (2 patients) in connection with fever episodes. These had onset on day of INTUVAX administration, the duration was short and all patients could be discharged the day after administration. Additionally, rash (1 patient) and hypotension (1 patient) were reported with onset just a few hours after administration. These drug-related adverse events were mild – moderate in terms of severity. No dose-limiting toxicity was found. SAEs reported between the first vaccine dose and nephrectomy were three occasions of pain; chest, musculoskeletal and abdominal, and one occasion of vertigo. SAEs reported after nephrectomy were one procedureal complication after nephrectomy (patient 7), one occasion of fever due to pneumonia and one occasion of dyspnoea (patient 10), one occasion of dizziness 2 months after first vaccination (patient 11), one occasion of pulmonary embolism 4 months after first vaccination (patient 12) and one occasion of renal failure 9 months after the first intratumoral administration of INTUVAX (patient 6). None of the SAEs reported were assessed as related to INTUVAX.

**Table 2:** Treatment-related adverse events possibly or probably related to INTUVAX after first and second vaccination

Reported Adverse event	No of related AEs (no. of patients)	CTCAE-grade (range)
Fever	8 (5)	1–2
Chills	2 (2)	1
Rash	1 (1)	1
Hypotension	1 (1)	2

The patients were also extensively monitored in terms of laboratory assessments, and abnormalities in haematology, biochemistry, coagulation, and renal function, were in most cases assessed as minor without any signs of INTUVAX inducing clinically significant changes. These minor abnormalities and also the more extensive changes (e.g. albumin and calcium levels at CTCAE 3–4 for patient 8 and 11, respectively) were seen as a result of their current disease.

### Tracking of injected INTUVAX cells

FACS analysis for donor (INTUVAX) cell tracking was performed after lysis of the erythrocytes on peripheral blood samples (taken just before vaccination and 1 h and 24 h after each vaccination), by staining with antibodies specific for the HLA class I antigens HLA-A2 or HLA-A24 that were selectively expressed on INTUVAX cells. The detection level for cell tracing was 0.0001% out of total circulating mononuclear cells (monocytes + lymphocytes) in blood. No tracking could be performed on patient 11 and 12, because the HLA-A tissue type of these two patients was A2 and A24 (thus identical to INTUVAX cells as to HLA-A tissue type). A measurable increase, corresponding to 8–35% of injected cells, was seen in the circulation 1 h and/or 24 h after administration in 4 out of 10 evaluated patients (patient 3, 8, 9 and 10).

### HLA-compatibility between donor and recipient

The tissue HLA-type of the INTUVAX cells was HLA-A:02,24, B:15,44 and DRB1:01,04. The HLA types A2, A24 and B44 are very frequent in the general Swedish population and approximately 40%, 15% and 30% of normal blood donors have the A2, A24 or B44 tissue type, respectively. When compared to the treated patients, none of the patients exhibited a fully mis-match (not expressing any of the INTUVAX HLA-molecules). Five patients exhibited 1 HLA-match, 5 patients exhibited 2 HLA-matches and 2 patients exhibited 3 HLA-matches (Table 3). No correlation between degree of HLA-match/mis-match and fever reactions or intratumoral infiltration of CD8+ T cells was found.

**Table 3:** HLA-type for donor and patients

HLA-type	HLA-A	HLA-B	HLA-DR
Donor	A2,24	B15,44	DR1,4
Patient 1	A2	B51,60	DR13
Patient 2	A2	B15,27	DR3,4
Patient 3	A2,3	B8,44	DR3,4
Patient 4	A3,32	B7,44	DR12,15
Patient 5	A1,29	B44	DR7,13
Patient 6	A2,11	B7,60	DR4,13
Patient 7	A26,32	B51,62	DR4
Patient 8	A1,24	B8,18	DR3,13
Patient 9	A2,3	B7,51	DR1,15
Patient 10	A24,29	B35,44	DR7,13
Patient 11	A2,24	B27,51	DR11,12
Patient 12	A2,24	B7	DR4,5

### Auto- and allo-immunization

No serological or clinical signs of induced autoimmunization by INTUVAX was observed, while 3 patients developed donor (INTUVAX) - specific alloimmunization (Table 4).

**Table 4:** Summary data for detected HLA antibodies and development of alloimmunization in patients

Patient	Dose (× 10^6^ cells)	HLA antibodies (baseline)	Donor-specific HLA antibodies (Day 120)
1	5	Present	No
2	5	Not present	No
3	5	Not present	No
4	5	Not present	Yes (Anti-DR4)
5	10	Not present	No
6	10	Present	No
7	10	Present (Anti-A2)	Yes (Anti-A2)
8	10	Not present	Yes (Anti-B44)
9	10	Present	Yes (Anti-A24,B44)
10	20	Present	Not done
11	20	Not present	No
12	20	Present	No

### Lymphocyte distribution and absolute number in peripheral blood

No significant changes in distribution or absolute number of CD3, CD4 or CD8 positive T cells, CD19+ B cells, CD3–16 + 56+ NK cells, CD3 + 16 + 56+ NKT cells or activated T cells (CD3 + HLA-DR+), NK cells (CD3–16 + 56 + 69+) or NKT cells (CD3 + 16 + 56 + 69+) were found 1 or 14 days after each administration of INTUVAX compared to base-line values before the first administration (data not shown).

### Serological immune parameters

Inflammatory immune parameters in peripheral blood, taken just before INTUVAX administration and 24 h after each administration, were analysed in order to evaluate potential systemic release or induction of relevant cytokines, chemokines and other inflammatory mediators after intratumoral administration of INTUVAX. However, none of the evaluated inflammatory parameters in peripheral blood, including CRP, chemokines and cytokines exhibited any significant increase or decrease after vaccination (data not shown).

### Tumor immunohistology

**Fig. 1:**
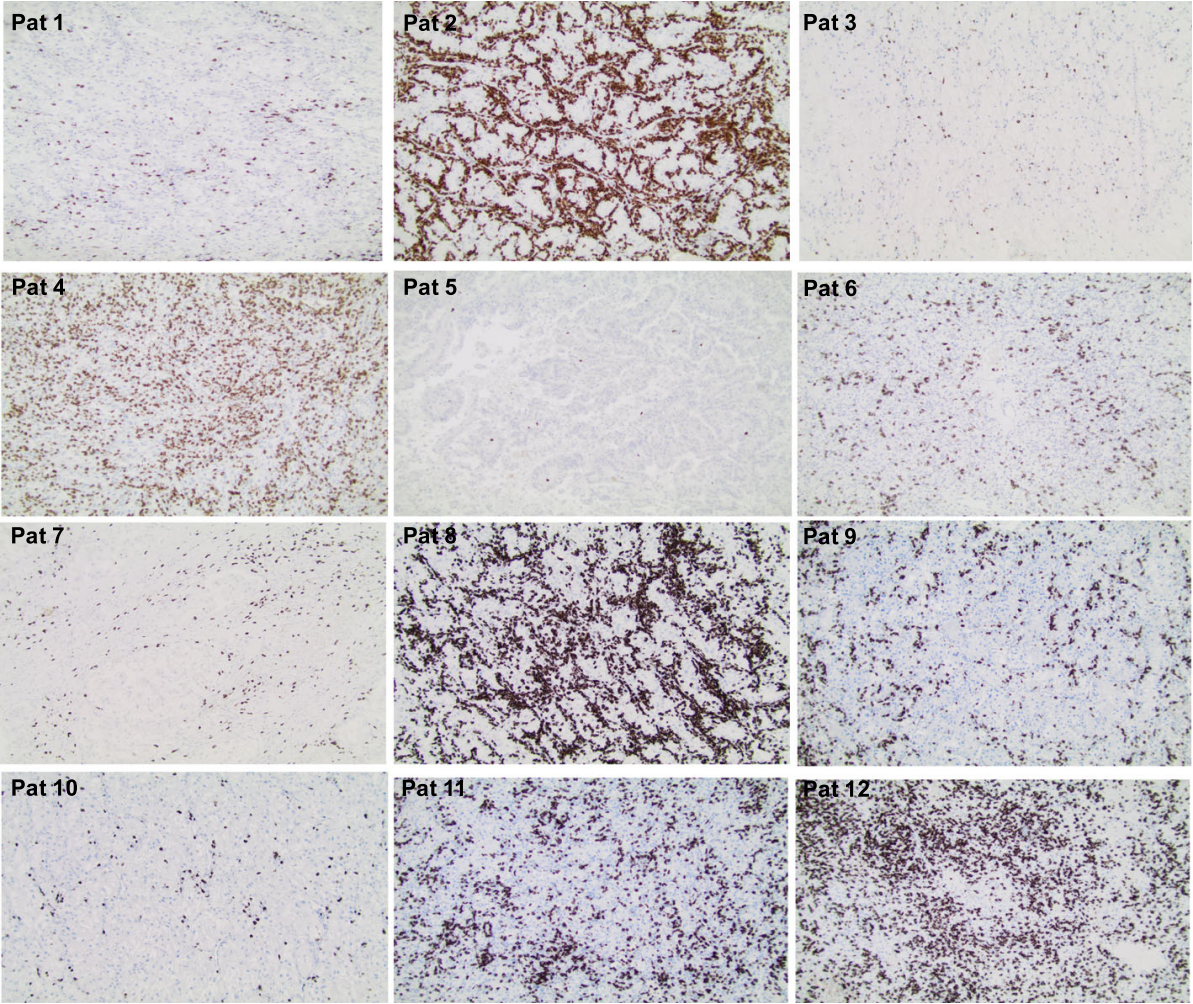
Micrographs illustrating immunohistochemical staining of tissue samples from all 12 surgically removed primary renal RCC tumors with anti-CD8 antibodies (see Methods). Original magnification × 100

Examined tumor tissues from patient 2, 4, 8, 11 and 12 exhibited a massive infiltration (median > 200 cells/high power field, × 400 magnification) of CD8+ T cells (Fig. 1 and Table 5). The tumor tissue from patient 6 and 9 exhibited a strong infiltration (median 50–199 cells/high power field, repectively, while the tumor tissue from the other 5 patients exhibited a moderate (median < 20–49 cells/high power field) or weak (median < 20 cells/high power field) infiltration (Fig. 1 and Table 5). The infiltration of CD8+ T cells in 3 randomly selected tumors from RCC patients that were not involved in the study, was also assessed and all three samples exhibited a low to moderate number (< 50 cells/high power field) of infiltrating CD8+ T cells (data not shown).

**Table 5:** Intratumoral CD8+ T cell infiltration

Patient	CD8/HPF	Grade of CD8 infiltration
1	44	Moderate
2	>200	Massive
3	33	Moderate
4	>200	Massive
5	<5	Weak
6	73	Strong
7	47	Moderate
8	>200	Massive
9	132	Strong
10	30	Moderate
11	>200	Massive
12	>200	Massive

CD8/HPF, median number of intratumoral CD8+ T cells/high power field (×400)

**Fig. 2:**
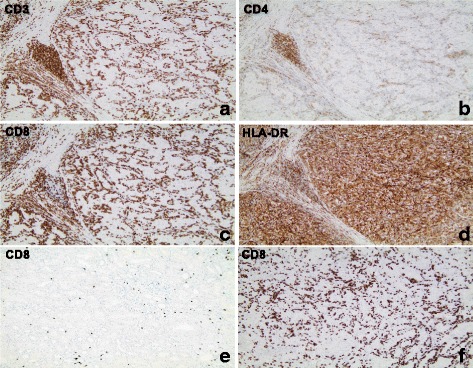
Micrographs illustrating immunohistochemical staining of tissue samples from surgically removed primary renal RCC tumors in consecutive sections from one RCC tumor with massive CD8+ T cell infiltration (**a**-**d**, all from patient 2). (**e**) illustrates CD8+ T cells in normal kidney parenchyma adjacent to tumor areas from patient 2 and (**f**) illustrates CD8+ T cell infiltration in a subcutaneous metastatic lesion from patient 2. Orignial magnification × 100

As illustrated in Fig. 2, the number and infiltration pattern of CD8+ T cells were nearly identical to the number and infiltration pattern of CD3+ T cells within tumor cell nests and by far outnumbered CD4+ T in tumor samples with high intratumoral T cells infiltration. In contrast, CD4+ T cells generally predominated in the stroma surrounding tumor cell nests (Fig. 2b). The normal kidney tissue areas adjacent to tumor areas, including those adjacent to tumor areas with high CD8+ T cell infiltration, were generally only weakly infiltrated with CD8+ T cells (Fig. 2E). In the only evaluable metastatic lesion surgically removed (subcutaneous lesion removed 1 month after nephrectomy) from a patient with massive CD8+ T cell infiltration in the primary kidney tumor, a similar massive infiltration of CD8+ T cells was seen (Fig. 2F).

**Fig. 3:**
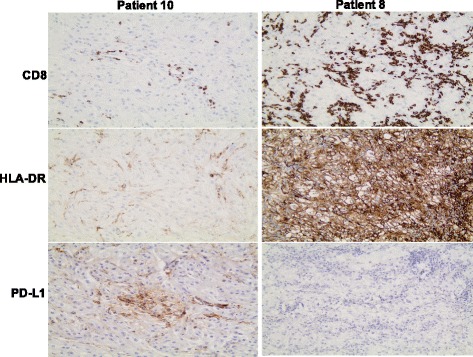
Micrographs illustrating immunohistochemical staining of tissue samples from two surgically removed primary renal RCC tumors with antibodies against CD8, HLA-DR and PD-L1 in consecutive sections. Original magnification × 200

Only a few scattered CD56+ NK cells were found in the tumor tissue from all 12 patients. CD68+ macrophage-like cells were usually seen scattered between the tumor cell nests (data not shown). All tumor samples, except the tumor from patient 5 (with a papillary RCC), exhibited at least some areas of HLA-DR-expressing tumor cells. A general and very strong HLA-DR expression in tumor cells was found in tumors from patient 2, 8 and 12, all three with a concomitant massive infiltration of CD8+ T cells (Figs. 2 and 3). Staining for PD-L1 expression revealed that only one out of six evaluated tumors (patient 2, 4, 8, 10 and 12) exhibited a positive staining that exceeded 5% of all tumor cells. Notably, this tumor (from patient 10) was characterized by a low CD8-infiltration and weak or absent HLA-DR expression on tumor cells (Fig. 3). In contrast, none of the analysed tumors with massive CD8 infiltration and with a concomitant strong HLA-DR expression (patient 2, 4, 8,11 and 12) showed no or less than 5% of PD-L1 expression in tumor cells (Fig. 3).

### Tumor-specific ELISpot

**Fig. 4:**
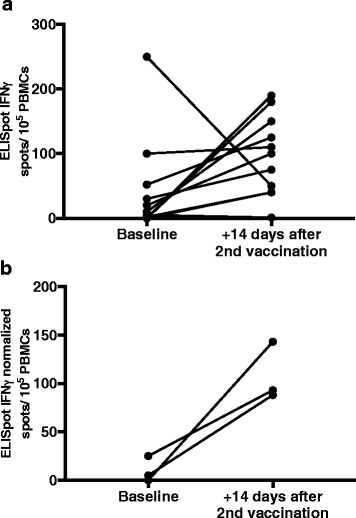
Tumor-specific and IFN-g producing peripheral blood lymphocytes measured by ELISpot at baseline and 2 weeks after the second ilixadencel dose. A direct ex vivo interferon gamma ELISpot analysis was performed and number of spots were counted. Samples were analyzed in triplicates, and mean response was calculated. In **a** baseline values are compared with values obtained 14 days after second administration and are shown for 9 out of 11 evaluated patients whereas in (**b**), baseline values and values obtained 14 days after second administration have been normalized to a negative control sample and are shown for 3 of the evaluated patients

Nine out of 11 evaluated patients exhibited an increased number of tumor-specific and IFN-γ producing lymphocytes in peripheral blood, as determined by ELISpot, when comparing baseline values with values obtained 2 weeks after the second administration of INTUVAX (Fig. 4a). However, in ELISpot samples from 8 out of 11 evaluated patients (six with increased numbers post-administration and two with decreased numbers post-administration), the negative control sample (without addition of autologous tumor cell lysate) exhibited a number of spots that a least at one time-point exceeded the numbers found when the autologous tumor cell lysate was added. Taken together, formally reliable ELISpot results was only present in three patients, and these three exhibited an increased number of tumor-specific and IFN-γ producing lymphocytes 2 weeks after the second INTUVAX administration as compared to base-line levels (Fig. 4b).

### Efficacy

Tumor response, as measured by comparing CT scan 3 months after nephrectomy with baseline by investigator assessment, according to RECIST 1.1 was evaluated in 8 patients. Patient 5 (incomplete metastasectomy during nephrectomy), patient 6 (myeloma lesions), patient 10 (deceased before the 3 months evaluation), patient 11 (not performed) were not evaluable. None of the 8 evaluable patients exhibited an objective tumor response, while 3 patients (patient 1, 2, 3) had stable disease and 5 patients (patient 4, 7, 8, 9, 12) had progressive disease.

### Follow-up with standard of care

The study was formally completed in March 2014, but the patients have thereafter been followed-up for tumor progression, subsequent systemic treatment and tumor response to these treatment regimes and survival time.

#### Subsequent treatment with sunitinib

**Fig. 5:**
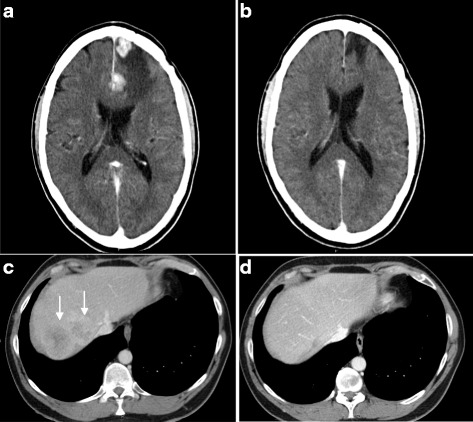
CT-scans from a patient with CNS and liver metastases 4 months after start of INTUVAX treatment but before additional treatment with sunitinib (**a** and **c**, respectively) and 6 months (**b**) or 12 months (**d**) after start of sunitinib treatment

Two of 3 patients, who so far have received subsequent therapy with sunitinib as first-line TKI treatment, have shown an objective tumor response (Fig. 5), and both responding patients exhibited a strong/massive infiltration of CD8+ T cells in their removed kidney tumor. Both patients had a MSKCC poor prognosis profile at inclusion and both received two doses of 10 million vaccine cells. One of these 2 patients (patient 8) exhibited sarcomatoid histological features in the resected primary tumor and the other patient (patient 9) had developed 4 brain and 3 liver metastases at the follow up visit 3 months after nephrectomy. Notably, the patient with brain and liver metastases responded with a complete disappearance of all brain and liver lesions (Fig. 5) and this response was still ongoing 38 months after initiation of sunitinib treatment.

The third patient (patient 12), who received subsequent treatment with sunitinib, exhibited a strong/massive intratumoral infiltration of CD8+ T cells experienced a stable disease for 6 months on sunitinib, but progressed upon discontinuation (due to unacceptable side effects) of sunitinib treatment, despite initiation of axitinib treatment. This patient had an MSKCC poor prognosis profile at inclusion and received 2 doses of 20 million vaccine cells.

#### Subsequent treatment with pazopanib

Among 3 patients receiving additional treatment with pazopanib as first-line TKI treatment, one (patient 1) has experienced an objective tumor response and another patient (patient 2) experienced a long-lasting (19 months) progression-free survival (PFS), after initiation of pazopanib treatment. The third patient (patient 3) deceased due to a profuse gastric bleeding 6 weeks after initiation of pazopanib treatment.

#### Subsequent treatment with immune checkpoint inhibitors

**Fig. 6:**
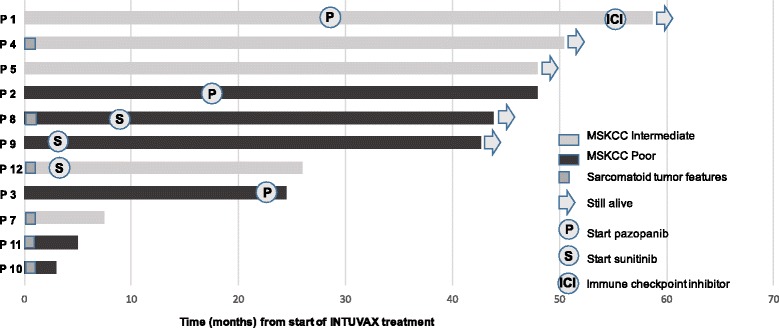
Swimmer plot with each bar representing the survival time from start of INTUVAX treatment for one patient

One patient (patient 1) has recently received immune checkpoint inhibitor (ICI) treatment. The patient started with nivolumab treatment 57 months after start of INTUVAX (28 months after start of subsequent TKI treatment) (Fig. 6). Follow-up CT scan 2 months after nivolumab start showed stable disease.

#### Subsequent treatment with surgery

In two patients, nephrectomy was followed by metastasectomy, one complete (patient 4) and one incomplete (patient 5). A subsequent complete tumor regression, without addition of any systemic treatment, was observed in the patient with incomplete metastasectomy, but a local recurrence was noted 18 months later. Patient 5 is still tumor free, 46 months after radical metastasectomy.

Three patients, all with sarcomatoid tumor features and a rapidly progressing mRCC, deceased within 7.5 months after the first INTUVAX dose and did only receive palliative treatment.

### Overall survival data

Updated survival data (06 December 2016) on the whole patient group (poor + intermediate risk) showed that five of the eleven patients evaluable for efficacy were still alive. Median OS for the entire patient group was 42.5 + (yet not reached) months. For the MSKCC poor risk sub-group (6 patients), the median OS was 32.8 + months and for the subgroup of patients with MSKCC intermediate risk (5 patients), the median overall survival was 47.8 + months.

## Discussion

INTUVAX is a cell-based immune enhancer/adjuvant that introduces the concept and possibility of using pre-produced and freeze-stored allogeneic human monocyte-derived DCs as an off-the-shelf product for therapeutic vaccination against cancer when injected intratumorally. The product is composed of pro-inflammatory DCs that have been stimulated with a combination of potent activating factors, resulting in a residual strong production of desirable DC-recruiting and DC-activating factors at the time of injection. The local release of these factors within the tumor is expected to induce a local recruitment and activation of endogenous immune cells, including natural killer (NK) cells, immature DCs and T cells. By using allogeneic DCs as vaccine cells, such cells will further be regarded as MHC-incompatible foreign invaders, most likely potentiating an inflammatory reaction that corresponds to an allogeneic mixed leucocyte reaction (MLR), further promoting recruitment and activation of endogenous DCs at the vaccination site [14]. Finally, engulfed tumor-derived proteins, including tumor-specific neoantigens within the injected target tumor tissue, are expected to act as antigen source for recruited endogenous DCs. The “final” outcome is that recruited endogenous DCs will capture dying tumor cells (killed by recruited and locally activated NK cells), and subsequently differentiate into T helper 1 deviating DCs. These DCs will finally migrate to the tumor-draining lymph node, where they will prime tumor-specific T cells, including cytotoxic CD8+ T cells.

The safety profile in this first-in human clinical trial on newly diagnosed mRCC patients showed that INTUVAX was well tolerated. In terms of AEs, the related events reported were few, and mild to moderate in terms of severity.

In terms of immunologic safety parameters, there were no signs of any induced autoimmune reactions, as measured by standard serological tests, while a humoral donor-specific alloimmunization was seen in 3 patients. In terms of immunologic efficacy parameters, 5 out of 12 resected tumors exhibited a massive infiltration (> 200 cells/high power field) of CD8+ T cells, which compares favourably with historical data where a median of approximately 20 CD8+ T cells/high power field was reported among 105 examined RCC samples [16].

No clear-cut correlation between number of infiltrating CD8+ T and the DC dose (5–20 million cells/dose), degree of MHC-incompatibility between vaccine cells and recipients or fever reactions was however found.

Therapies targeting the programmed death-1 (PD-1) receptor have shown unprecedented rates of durable clinical responses in patients with various cancer types, including mRCC [18]. One mechanism by which cancer tissues limit the host immune response is via upregulation of PD-1 ligand (PD-L1) and its ligation to PD-1 on antigen-specific CD8 T cells. PD-L1 can be constitutively expressed on the surface of cancer cells through poorly characterized oncogenic signalling pathways [19, 20] or alternatively, expressed in response to the presence of CD8+ T cells, producing immune-stimulating cytokines, including IFN-γ [21]. The latter process has been termed adaptive immune resistance [22] and represent a mechanism by which cancer cells attempt to protect themselves from immune cell-mediated killing. In line with this adaptive immune resistance process, findings by Tumeh and collegues [21] indicate that melanoma tumor regression, following therapeutic PD-1 blockade requires pre-existing CD8+ T cells. Our present data, as to PD-L1 expression, are however highly surprising because strong CD8+ T cell infiltration, which was generally associated with strong HLA-DR expression on adjacent tumor cells (potentially indicating the presence of CD8-derived IFN-γ) was associated with the abscence of, or low, PD-L1 expression in tumor cells. In contrast, the tumor with substantial expression of PD-L1 was one with poor CD8+ T cell infiltraton and concomitant low or absent expression of HLA-DR expression. These finding are, however, at least partially in line with a recent publication where no correlation was found between PD-L1 tumor expression and IFN-γ or cytotoxic T cell gene signatures in RCC, while melanoma tumors exhibited the expected correlation [23]. Taken together, these data may indicate that RCC expression of PD-L1 is mainly regulated by oncogenic signalling pathways.

In terms of clinical efficacy, none of the patients in our mRCC cohort exhibited any objective tumor response at 3 months, while the majority (five out of 8) of evaluable patients actually exhibited a tumor progression, according to RECIST 1.1. criteria.

The study was formally completed in March 2014, but the patients have thereafter been followed as to tumor progression, subsequent systemic treatment, tumor response to these treatment regimens and survival time. Three patients, all with sarcomatoid tumor features and with a rapidly progressing mRCC, only received palliative care after nephrectomy. Two of 3 patients, who so far have received subsequent therapy with sunitinib, have shown an objective tumor response and both responding patients exhibited a strong/massive infiltration of CD8+ T cells in their removed kidney tumor. One of these 2 patients exhibited sarcomatoid tumor features and the other patient had developed 4 brain metastases at the follow up visit 3 months after nephrectomy. Notably, the patient with brain metastases responded with a complete disappearance of all brain lesions and this response was still ongoing 38 months after initiation of sunitinib treatment. These two cases of objective tumor response upon subsequent sunitinib treatment are highly unexpected, since recent publications indicate that brain metastases as well as mRCC with sarcomatoid features are highly resistant to sunitinib treatment [24, 25]. These findings therefore indicate that INTUVAX given in conjunction with sunitinib may have a synergistic antitumor effect. Our hypothesis is that INTUVAX elicit a tumor-specific T cell response and that addition of sunitinib, which has been shown to affect and reduce the number of myeloid-derived suppressor cells and regulatory T cells [26, 27], will unleash the tumor killing potential of INTUVAX-induced tumor-specific T cells. A synergistic vaccine/sunitinib combination effect has been observed in a mouse model and supports this hypothesis [28]. Among 3 patients, receiving additional treatment with pazopanib, one has experienced an objective tumor response and another patient experienced a long-lasting (19 months) progression-free survival, after initiation of pazopanib treatment. In two patients, nephrectomy was followed by metastasectomy, one complete and one incomplete. A subsequent complete tumor regression, without addition of any systemic treatment, was observed in the patient with incomplete metastasectomy, but a local recurrence was noted 18 months later. Patient 5 is still tumor free, 46 months after radical metastasectomy. These data compares favourable with historical data from a group of 22 patients with mRCC, receiving targeted therapy followed by complete surgical resection of metastatic disease [29]. These patients developed tumor recurrence at a median time of 10.5 months from metastasectomy.

Updated survival data (06 December 2016) for the whole patient group is, moreover, highly encouraging, showing that 5 of the 11 patients evaluable for efficacy were still alive. Median OS (from start of INTUVAX treatment) for the entire patient group was 42.5 + (yet not reached) months as compared to expected 14.7–15.8 months mOS for poor and intermediate risk patients with newly diagnosed (treatment initiation within one year from diagnosis) mRCC receiving standard targeted therapy [30, 31]. In a recent publication on newly diagnosed mRCC patients with poor and intermediate prognosis (48% with poor prognosis according to IMDC criteria) treated with autologous DCs transduced with autologous tumor mRNA in combination with sunitinib, a mOS of 30.2 months was reported [32]. Moreover, in a recent publication by Choueiri and collegues [33] on patients with poor and intermediate mRCC prognosis a mOS of 30.3 months was reported for cabozantinib, as compared to 21 months for sunitinib. However, only 19% of the patients in the cabozantinib and sunitinib groups belonged to the high risk/poor prognosis subgroup (according to IMDC criteria) while 45% of our patients belonged to this IMDC subgroup.

There is only few historical data available as to mOS in the separate subgroup of newly diagnosed mRCC patients with high risk [32, 34]. In the study by Powles and collegues [34] the mOS for high risk patients, according to MSKCC criteria, was 9.0 months as compared to 32.8+ months in our MSKCC high risk subgroup. In the study by Amin and collegues [32], the mOS for the high risk subgroup, according to IMDC criteria, was 9.1 months, as compared to 24.5 months in our IMCD high risk subgroup.

High base-line density of CD8+ T cells has been shown to be a good prognostic feature in a wide variety of solid tumors [35]. In RCC, however, patients with highest intratumoral CD8+ T cell density have repeatedly been shown to have shorter survival [36, 37]. In contrast to these historical RCC data, mOS for those 5 patients in our mRCC cohort with massive infiltration (> 200 cells/high power field) is currently superior compared to mOS for the other 6 patients with low or moderate CD8+ T cell density (44.1+ months vs. 33.5+ months). However, mOS in our subgroup of patients with low or moderate CD8+ T cell density also compares favourable with historical mOS data and 2 out of 3 patients with the currently longest survival time belongs to the subgroup with weak or moderate intratumoral CD8+ T cell infiltration.

## Conclusion

Taken together, these data indicate that intratumoral injection of INTUVAX is safe and induce a systemic antitumor immune response that may prolong survival in mRCC patients subsequently receiving standard of care. These encouraging findings support the ongoing phase 2, randomized, multi-centre, MERECA study (Clinicaltrials.gov identifier NCT02432846), which has been designed to compare the addition of INTUVAX with standard surgery and sunitinib therapy to standard surgery and sunitinib alone in newly diagnosed mRCC patients.

## Abbreviations

CTC: Common Toxicity Criteria
CTL: Cytotoxic T cell
DC: Dendritic cell
ECOG: Eastern Cooperative Oncology Group
ELISpot: Enzyme-Linked Immunosorbent Spot
GM-CSF: Colony-stimulating factor
IFN-γ: Interferon gamma
MLR: Mixed leucocyte reaction
mOS: Median overall survival
mRCC: Metastatic renal cell carcinoma
NK cell: Natural killer cell
PBMCs: Peripheral blood mononuclear cells
PD-1: Programmed cell death protein
PD-L1: Programmed cell death ligand
RECIST: Response Evaluation Criteria In Solid Tumors
TKI: Tyrosine kinase inhibitor
TLR: Toll-like receptor
